# Low-Cost, Real-Time Polymerase Chain Reaction System with Integrated RNA Extraction

**DOI:** 10.3390/s23104604

**Published:** 2023-05-09

**Authors:** Tchamie Kadja, Yvonne Sun, Vamsy P. Chodavarapu

**Affiliations:** 1 Department of Electrical and Computer Engineering, University of Dayton, 300 College Park, Dayton, OH 45469, USA; kadjat1@udayton.edu; 2 Department of Biology, University of Dayton, 300 College Park, Dayton, OH 45469, USA; ysun02@udayton.edu

**Keywords:** polymerase chain reaction (PCR), fluorescence sensing, real-time PCR, RNA extraction, point-of-care diagnostics, COVID-19, food and water quality

## Abstract

Rapid, easy-to-use, and low-cost systems for biological sample testing are important for point-of-care diagnostics and various other health applications. The recent pandemic of Coronavirus Disease 2019 (COVID-19) caused by the Severe Acute Respiratory Syndrome Coronavirus 2 (SARS-CoV-2) showed an urgent need to rapidly and accurately identify the genetic material of SARS-CoV-2, an enveloped ribonucleic acid (RNA) virus, in upper respiratory specimens from people. In general, sensitive testing methods require genetic material extraction from the specimen. Unfortunately, current commercially available extraction kits are expensive and involve time-consuming and laborious extraction procedures. To overcome the difficulties associated with common extraction methods, we propose a simple enzymatic assay for the nucleic acid extraction step using heat mediation to improve the polymerase chain reaction (PCR) reaction sensitivity. Our protocol was tested on Human Coronavirus 229E (HCoV-229E) as an example, which comes from the large *coronaviridae* family of viruses that affect birds, amphibians, and mammals, of which SARS-CoV-2 is a member. The proposed assay was performed using a low-cost, custom-made, real-time PCR system that incorporates thermal cycling and fluorescence detection. It had fully customizable reaction settings to allow versatile biological sample testing for various applications, including point-of-care medical diagnosis, food and water quality testing, and emergency health situations. Our results show that heat-mediated RNA extraction is a viable extraction method when compared to commercial extraction kits. Further, our study showed that extraction has a direct impact on purified laboratory samples of HCoV-229E, but no direct impact on infected human cells. This is clinically relevant, as it allows us to circumvent the extraction step on clinical samples when using PCR.

## 1. Introduction

The recent pandemic of the Coronavirus Disease 2019 (COVID-19) caused by the Severe Acute Respiratory Syndrome Coronavirus 2 (SARS-CoV-2) has had an unprecedented worldwide economic, social, and health impact. It is estimated that there have been more than 760 million cases of COVID-19 worldwide, with more than 8 million casualties [1]. Despite the increased number of vaccines administered to help alleviate the disease transmission and incidence, COVID-19 outbreaks still occur in different regions of the globe, partially due to continuous genetic mutations of the pathogen leading to evolution of new viral strains. Therefore, rapid and ubiquitous medical testing is crucial for surveillance that could help contain the spread of the pandemics [2,3].

Among various available means, polymerase chain reaction (PCR) systems are widely used for COVID-19 medical diagnostics. Currently, this technique is frequently used to analyze upper respiratory specimens from patients and allows the detection of the ribonucleic acid (RNA) of SARS-CoV-2. PCRs involve enzymatic amplification of a specific DNA sequence from a complex pool of genetic material [4,5]. PCR systems have many applications in environmental species monitoring, gene and pathogen analysis, or food quality examination [6,7,8,9]. PCRs are also extensively used for testing foodborne and waterborne diseases caused by pathogens, such as *Listeria monocytogenes*, *E. coli* O157:H7, *Salmonella*, and *Campylobacter* [10,11]. Unfortunately, due to the high-cost nature of PCR devices and the lack of properly trained personnel to operate medical equipment, resource-limited areas have limited access to proper medical testing.

To date, many low-cost PCR systems have been proposed, but many do not integrate real-time reaction monitoring with built-in fluorescence detection capabilities [12,13,14,15,16], which would require the use of time-consuming gel electrophoresis. Further, most real-world PCR biological assays require customized parameters tailored to the reaction in terms of cycle temperatures, number of steps, and hold times. To this end, we have previously described a cost-efficient, user-friendly, portable, and real-time PCR (qPCR) system that can be used for DNA amplification and genetic material quantification using fluorescence [17]. *Listeria monocytogenes*, a Gram-positive bacterial pathogen, was previously used as a test sample to assess the system performance, given its threats to food safety [18]. The system was compared to currently developed low-cost and portable PCR devices [17].

In this paper, we describe the testing of our low-cost qPCR system to handle samples with RNA-based pathogens, which requires the use of a reverse transcription step prior to DNA amplification. This shows the versatility of our system in handling both DNA- and RNA-type pathogens without requiring any hardware modification. In addition, our system aims to address the process of genetic material extraction from real-world collected samples, unlike most low-cost systems, which use external commercially available extraction kits. The extraction of DNA or RNA is crucial for pathogen detection, especially at low viral concentration, which would help to improve accuracy of results and reduce false negatives [19,20]. Having an integrated extraction method would therefore be beneficial in terms of reaction sensitivity, reaction cost, and reaction duration. Commercially available DNA or RNA extraction kits are laborious and time-consuming when it comes to achieving good extraction efficiency [19]. Previously, many genetic material extraction protocols have been assessed in terms of efficiency and complexity [21,22,23,24,25,26,27,28,29]. Most of these protocols employ chemical or enzymatic lysis solutions (organic solvent or detergent), mechanical disruption, magnetic separation, freeze–thaw breakage, heat RNA-release, or a combination of several methods. A survey of those protocols showed that these methods can be expensive, complex, and time-consuming [22,23,24,25]. Heat-release of genetic material was found to be the most convenient method [29]. To this end, we propose an integrated heat-assisted RNA extraction method with our low-cost reverse transcription PCR (RT-PCR) system. Human Coronavirus strain 229E (HCoV-229E) is a member of the *Coronaviridae* family of enveloped viruses and was used as target sample in our work. Unlike the pandemic strain, SARS-CoV-2, HCoV-229E is known to cause mild respiratory illness. This manuscript is organized as follows: in Section 2, we present an overview of the materials and methods, followed by results and discussion in Section 3 and Section 4. Finally, we provide brief conclusions of our study in Section 5.

## 2. Materials and Methods

### 2.1. System Architecture

**Figure 1:**
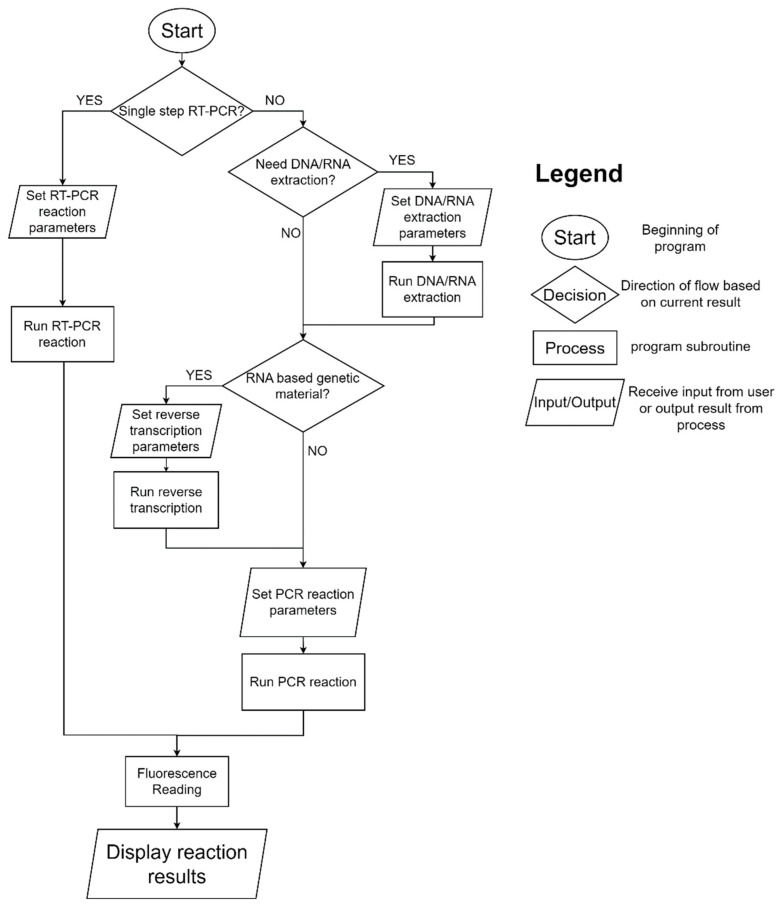
Flowchart of system parameterization.

**Figure 2:**
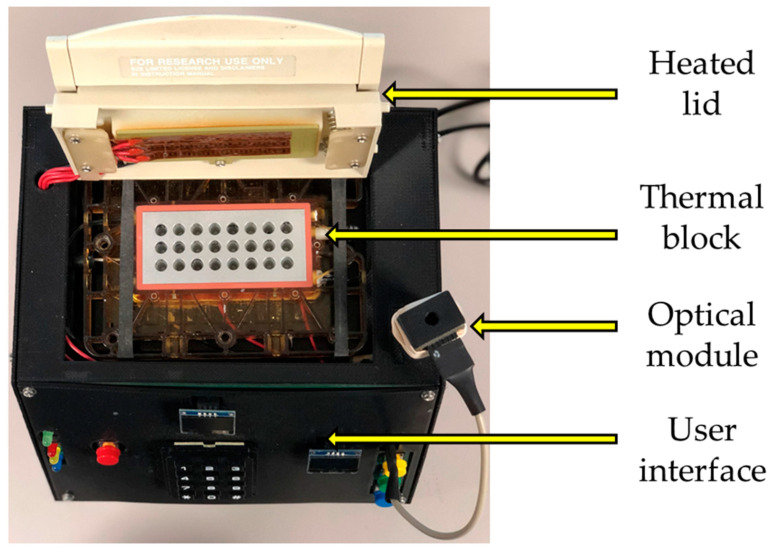
Exterior appearance of the system.

The architecture of our RT-PCR system was described in a previous publication [17]. Briefly, it is comprised of a control unit integrating an ATmega2560 microcontroller (Microchip Technology, Chandler, AZ, USA) an optical system using an AS7341 spectrometer (AMS, New York, NY, USA), a communication module housing an ESP8266 Wi-Fi chip (Espressif Systems, Shanghai, China) and some input/output devices. Because of the addition of the RNA-based pathogens assays, the system software was updated, adding the functionalities of DNA/RNA extraction and reverse transcription as part of the system user interface. This update was performed without requiring any hardware alterations. The flowchart of our current system parameterization is shown in Figure 1. The system can carry both single-step and multiple-step RT-PCR reactions. The exterior appearance of the system is displayed in Figure 2, and the system components were described previously [17].

### 2.2. Gel Electrophoresis Analysis

The qualitative analysis of the outcome of some PCR reactions was performed using a 1% or 1.2% agarose gel (wt/vol in Tris-Borate-EDTA buffer) prepared for the experiment. The gel electrophoresis was run at 100 volts for 25 min. Samples for gel electrophoresis were prepared by mixing 1 µL of loading dye (MaestroSafe, cat. no. MR-031201) and 10 µL of PCR sample. A DNA ladder (Accuris, cat. no. PR4100-100), loaded at 5 µL per lane, was used as a size standard. The resulting gel images were captured using a UVP Multispectral Imaging System (Biospectrum 500, LM-26, BioChemi 500 Camera f/1.2).

### 2.3. Heat-Assisted Extraction Protocol

The destabilization of the structural integrity of a pathogen from heating could result in the release of its genetic material, as suggested by prior literature [28], but the temperature at which such alteration occurs depends on the type of pathogen, as well as the physicochemical conditions, such as the additional use of magnetic beads, detergent, and lysis buffer [28]. RNA generally degrades more rapidly than DNA in a manner sensitive to factors such as time and temperature [30]. In some cases, RNA exposure to heat at an inappropriate temperature and duration could be detrimental to the reaction, presumably due to breakage of phosphodiester bonds within the targeted genetic material sequence [28,29]. To assess the effect of RNA extraction on RT-PCR sensitivity, HCoV-229E, an enveloped RNA virus, was obtained (American Type Culture Collection, VR-740, Manassas, VA, USA) and used in this work as a target sample. Two different viral samples were tested in this study. First, the virus was obtained as cryopreserved infected human fibroblast MRC-5 cells (ATCC, VR-740, VA, USA). Based on the manufacturer’s information, the HCoV-229E present in this sample includes both intracellular viruses undergoing replication and virions released in the culture supernatant from the infected cells. Second, HCoV-229E was propagated in MRC-5 cells (ATCC CCL-171) at 30 °C in DMEM (Corning™ 10013CV) supplemented with FBS (10%, *v*/*v*). The stock solution was then prepared by collecting the supernatant of infected MRC-5 cells (at 5–7 days post infection) and centrifuging the supernatant at 2000 rpm for 5 minutes to remove cellular debris. The resulting cell-free viral stock solution was aliquoted and stored at −20 °C.

**Figure 3:**
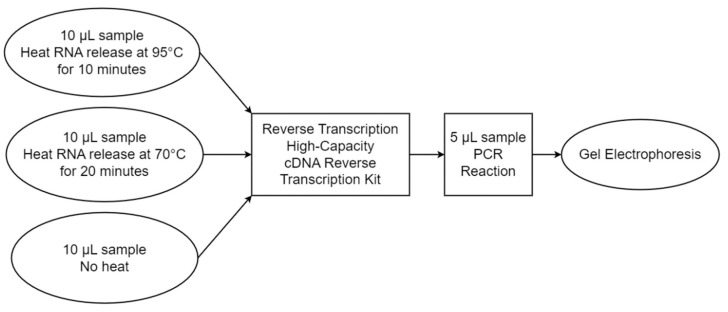
Heat extraction protocol assessment flowchart.

The initial step was to develop a proper RNA extraction protocol before carrying out the RNA detection assay on the RT-PCR system. Two factors, temperature and exposure time, were combined and applied for the RNA extraction on both the infected MRC-5 cell culture (CC) and the cultured viral solution (CV), and the extraction efficiency was monitored via gel electrophoresis. The two combinations used were 70 °C extraction for 20 min or 95 °C extraction for 10 min, where no heat control was also included for the RT-PCR reaction. The extraction was performed using 10 µL of each CC and CV viral solution, as well as deionized water as a no template control (NTC). The reaction was also run simultaneously on a commercially available RT-PCR system, StepOnePlus (Applied Biosystems, model: StepOnePlus), as a comparison. The experiment was replicated three times to assess the reproducibility of the method. Figure 3 shows the flowchart of the heat extraction protocol experiment.

### 2.4. Reverse Transcription Conditions

Genetic material amplification on RNA-type viruses requires the synthesis of a complementary DNA (cDNA) sequence from RNA sequences prior to the PCR reaction. The reverse transcription step for this experiment was performed using the High-Capacity cDNA Reverse Transcription Kit (Applied Biosystems, ca. no. 4368814), with a set of custom-designed primers and 10 µL of the extraction solution as a template in a total reaction volume of 20 µL. The set of custom primers designed for this experiment targeted both the nucleoprotein (N) gene and the spike glycoprotein (S) gene of HCoV-229E. The two genetic sequences are particularly important in characterizing the HCoV-229E virus family [31]. Primer stock solutions were prepared at a 100 µM concentration and were each diluted to a final working stock solution of 20 µM. Table 1 gives details about the reaction setup.

The primers were designed to produce amplicon sizes around 600 bp based on the following rationale. It was determined experimentally that shorter amplicon sizes (379 bp and 354 bp) were subjected to contamination and false positives, while longer amplicon sizes (999 bp and 1004 bp) lead to false negatives with the PCR parameters used in this experiment.

The cDNA synthesis reaction protocol was as follows: 10 min primer annealing at 25 °C, followed by 120 min DNA polymerization at 37 °C, followed by 5 min enzyme deactivation at 85 °C. After the reaction, the resulting cDNA solution was used as input for the PCR step.

### 2.5. PCR Conditions

For PCR reactions, 5 µL of the cDNA solutions were used as the input, mixed with Select MasterMix (Applied Biosystems SYBR, Mfr. No. 4472908) and different primer sets (Table 1). The PCR protocol used was as follows: 5 min uracil-DNA glycosylase (UDG) activation at 50 °C, followed by 10 min hot-start at 95 °C, 40 cycles of denaturation at 95 °C for 15 s, annealing and extension at 60 °C for 60 s, and a final extension at 60 °C for 5 min. Table 1 summarizes the target solution type, the extraction parameters, the reverse transcription, and the PCR reagents, as well as the primer sequences used for the overall RT-PCR reaction. The products of the RT-PCR reaction were analyzed with gel electrophoresis.

### 2.6. Heat-Assisted RNA Extraction Efficiency Assessment

**Figure 4:**
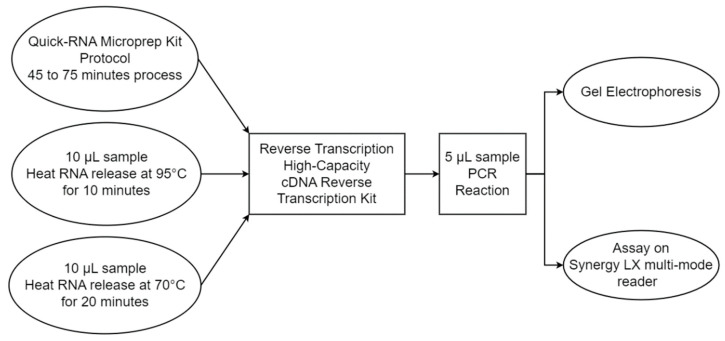
Extraction efficiency comparison between commercial kit and custom protocol flowchart.

The heat-assisted RNA extraction efficiency was compared to a commercially available, column-based RNA extraction kit (Quick-RNA Microprep Kit, Zymo, ca. no. R1050), following manufacturer’s protocol. The protocol is detailed in Appendix A of this report. A total of 10 µL of cultured cells or cultured viral solution was used for RNA extraction performed using heat mediation at 95 °C/10 min, heat mediation at 70 °C/20 min, and the column-based extraction method. The concentration of genetic material prior to and after extraction was measured using a Synergy LX multi-mode reader (BioTek Instruments, model: SLFXA, Santa Clara, CA, USA). We ran an RT-PCR reaction following the extraction step using both the nucleoprotein and the spike protein primers. The reaction progression was monitored using real-time fluorescence detection, and the final products were analyzed with gel electrophoresis as well. Beyond the extraction efficiency, the comparison between the heat-assisted and column-based extraction methods was also based off the number of steps, the complexity level, the runtime, and the overall cost. Figure 4 describes the comparison between the two protocols. The results of the comparison are discussed in subsequent sections of this paper.

### 2.7. RT-PCR Performance Comparison between the Custom PCR and Commercial Systems

We further compared the efficiency of our Custom RT-PCR system with a commercially available PCR system, StepOnePlus (System Version 2.2.3). The RT-PCR reaction was conducted concurrently, using both CC and CV as the starting samples. The extraction step was achieved using both 70 °C/20 min and 95 °C/10 min protocols on both devices. The same reverse transcription and PCR reaction steps were performed on both our custom RT-PCR and StepOnePlus systems, as described previously. While 0.1 mL tubes were used for the StepOnePlus system, 0.2 mL tubes were used in our custom system to allow better adhesion with the heated lid. All tubes were low-profile polypropylene tubes. The result was analyzed via gel electrophoresis. Our RT-PCR fluorescence measurement was compared with the commercial device Synergy LX multi-mode reader, with a green filter cube loaded (BioTek 1505005, Ex 485/20, M 510, Em 528/20).

## 3. Results

### 3.1. Heat-Assisted Extraction Protocol

**Figure 5:**
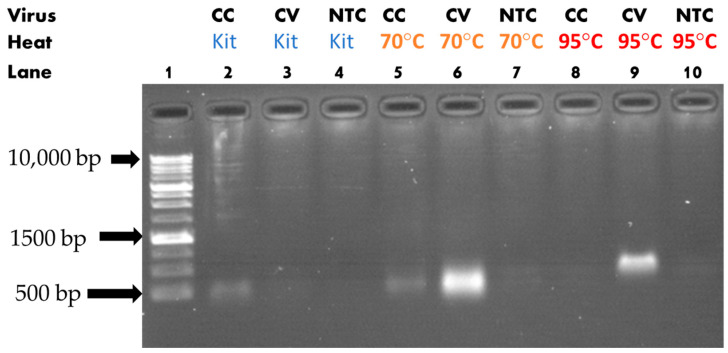
Gel electrophoresis results for nucleoprotein primer. CC, cell culture; CV, cultured viral solution; S, spike protein; N, nucleocapsid.

The gel electrophoresis following the PCR reaction showed that heat-assisted extraction can affect the PCR reaction sensitivity by several orders of magnitude. Figure 5 shows the casted agarose gel under UV illumination for the nucleoprotein primer assay. A slight difference in the band’s height was observed, possibly due to the non-uniformity of the gel concentration or due to a difference in DNA conformation. From Figure 5, in the case of CV, there was genetic material amplification only when heat was applied to the sample prior to the RT-PCR reaction, as lane six and nine showed luminescence, unlike lane three. It could be observed also that lane five (70 °C extraction) was brighter than lane eight (95 °C extraction). This could be explained by the adverse effect of high temperature on RNA preservation, as discussed earlier. A similar analysis could be performed when observing CC. In this case, heat had no direct positive effect on the sensitivity of the reaction, as lane two and five showed the same level of luminescence. Nevertheless, exposing the sample to a higher temperature was detrimental to the reaction, as lane eight showed less luminescence (no observable luminescence on the capture), an effect that was similar in the case of CV.

**Figure 6:**
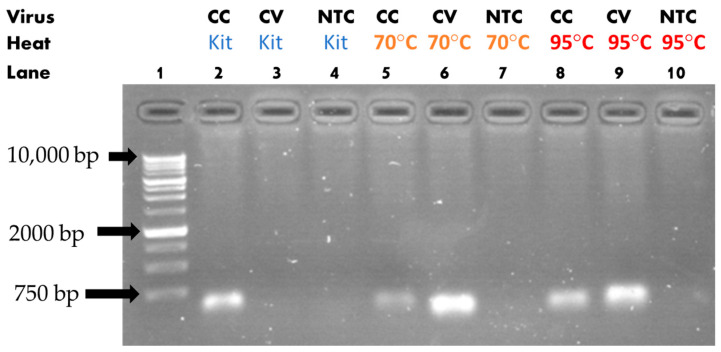
Gel electrophoresis results for spike protein primer.

The assay performed using the spike protein primer is displayed in Figure 6. As previously shown with the nucleoprotein primer, when observing CV, it could be seen that the heat extraction enabled the genetic material amplification, as lanes six and nine showed luminescence, unlike lane three. The extraction at 70 °C also showed brighter luminescence here as compared to the 95 °C extraction. The analysis for CC once again showed that extraction did not have a positive effect on the reaction sensitivity. The heat-exposed sample showed less luminescence, with 70 °C extraction being the least sensitive. This last result was different than our previous results, where 95 °C seemed to have the least sensitivity. The extraction efficiency for each extraction protocol was assessed and confirmed using fluorescence in multiple experiments, and the results are discussed in subsequent sections of this present paper.

### 3.2. Heat-Assisted RNA Extraction Efficiency Assessment

**Figure 7:**
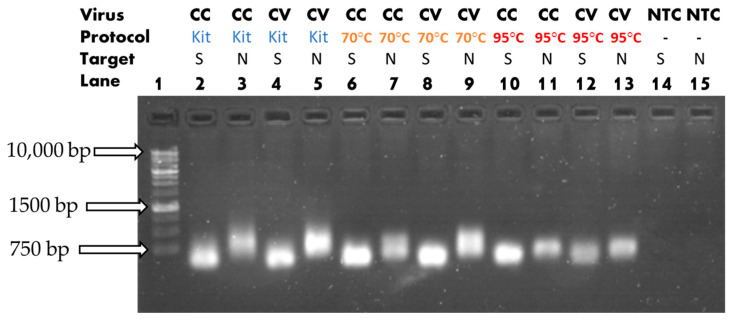
Gel electrophoresis results for all heat extraction protocols and RNA extraction kit. CC, cell culture; CV, cultured viral solution; S, spike protein; N, nucleocapsid.

We observed the product with gel electrophoresis after RT-PCR. The reaction employed heat mediation and a commercial kit on both CC and CV for RNA extraction. The result shows that there was successful amplification of the target in the case of both types of primers across all combinations, as illustrated in Figure 7. There was a noticeable difference in the luminescence brightness for the trios 2-6-10, 3-7-11, 4-8-12, and 5-9-13, which showed the luminescence levels of the extraction kit, 70 °C, and 95 °C, in that order. The extraction effectiveness of each combination was assessed and compared using an optical reader.

First, the RNA concentration level measurements prior to and after extraction were estimated from A260 values, and the results are compiled in Table 2. Such an experiment was replicated three times. The values were reported as means and standard deviations (SDs). The result shows an increase in RNA concentrations as calculated from A260 values after extraction using heat mediation. Nevertheless, because there was no purification in the case of heat extraction, there was a low genetic material purity index, as displayed by the A260/A280 ratio. The A260/A280 ratio describes the absorbance of a sample at 260 nm and 280 nm wavelengths. Nucleic acids absorb light at 260 nm and proteins absorb light at 280 nm. A ratio of 2.0 and above is generally considered as “pure” RNA (no protein contamination), and a ratio of 1.8 and above can be considered “pure” DNA. In comparison, the data showed a lower post-extraction RNA concentration level in the case of the Microprep Kit. Nevertheless, the A260/A280 ratio for the Microprep Kit was above 1.8 in most cases, indicating purer RNA presence. Therefore, for a more accurate comparison, the extraction efficiency should be analyzed during the PCR phase. By finding the Cycle Threshold (C_T_) values corresponding to each extraction protocol, the amount of targeted genetic material released during extraction could be compared between protocols.

The real-time monitoring of the PCR reaction that followed each extraction protocol was replicated three times. The C_T_ values for each sample type, extraction protocol, and primer combination are compiled in Table 3. The C_T_ values were reported as means and standard deviations. The results show that in almost all cases, the 70 °C/20 min extraction protocol had lower C_T_ values when compared to 95 °C/10 min, and that result confirmed the gel electrophoresis analysis. In our testing, the 95 °C/10 min protocol did not reach the C_T_ value within 40 cycles in one of the three experiments performed on the cultured cells and targeting the nucleoprotein. The other two C_T_ values were 36.46 and 37.02. Again, this confirmed the adverse effect of high temperature on genetic material integrity. The result also shows that the commercial kit provided an improvement when targeting the nucleoprotein, whereas it did not show noticeable improvement in the case of the spike protein.

### 3.3. RT-PCR Performance Comparison between Custom RT-PCR and Commercial Unit

Rapid specimen processing in practical applications will require circumventing the use of gel electrophoresis for result analysis. Real-time RT-PCR was run using both types of HCoV-229E samples on the commercial and custom PCR units. The final fluorescence measurement comparison between the commercial unit and our RT-PCR optical system is described in Table 4. The measurement values shown here were normalized for each system but were not normalized across both devices. The values used for the commercial unit were the normalized reporter values Rn. The results show that the fluorescence intensity was hindered by the presence of viral intra-cellular material such as proteins, since the nucleic acid extraction protocol did not include a purification step. This result was consistent between both devices with the PCR protocol used in this experiment. In addition, the recorded fluorescence intensity was dependent on the type of primer/sample pairing that was used. The fluorescence intensity was best on both devices when using the CV sample with the N gene primer. CV samples had higher viral particle concentration as compared to CC samples, and the current result was expected. Nevertheless, our custom RT-PCR system performed better by successfully detecting CV/spike primer templates, unlike the commercial unit, where the fluorescence reading was below the NTC threshold. CC samples had marginally low fluorescence reading across both devices, suggesting that low amplicon concentration templates should employ more than 40 cycles for the PCR protocol.

## 4. Discussion

Rapid, simplified, and low-cost infectious disease specimen testing is important to evaluate the dynamics of a pandemic or health crisis, especially in resource-poor areas of the world. Our study showed that heat mediation is a viable RNA extraction method that can be used in lieu of commercially available RNA extraction kits, although it could result in less efficiency, as seen with the delay in the CT value and the lower fluorescence intensity recordings for some target genes and sample types. This limitation can be compensated for by having additional PCR cycles. The overall experiment also showed that the 70 °C/20 min extraction protocol resulted in better PCR sensitivity with the test samples as compared to 95 °C/10 min, as RNA degrades more rapidly with time and temperature. Reverse transcriptase, the enzyme used in reverse transcription, is generally inactivated in the range of 70–85 °C [32]. This makes heat-mediated extraction suitable for single-step RT-PCR reactions if the extraction is optimized below 70° C.

With the samples used in the current study, our experimental results reveal that heat mediation was more useful on purified viral solution as compared to cultured infected human cells. This has clinical implications when using RT-PCR for medical diagnostics, as no heating of the collected infectious specimen is advised or necessary prior to the assay. The analysis also reveals that using the spike protein primer resulted in better PCR sensitivity on cultured infected human cells as compared to the nucleoprotein primer. Again, this has medical implications when using RT-PCR for medical diagnostics on the *coronaviridae* family of viruses, as they mostly share two envelope proteins, the membrane (M) and spike (S) proteins [33]. A similar result was found in recent studies on SARS-CoV-2 spike versus nucleoprotein antibody responses [34].

## 5. Conclusions

We described a low-cost, portable, and simplified pathogen assay system that is suitable for medical diagnostics, as well as food and water quality testing. Assays were successful on Human Coronavirus 229E, showing that suitable extraction can be achieved at lower temperatures such as 70 °C. The detection of the S protein had overall better sensitivity as compared to the N gene on the samples used in this experiment, a result that can be extended to the *coronaviridae* family of viruses as well. In practice, the testing chamber in the PCR system should be enclosed to prevent negative effects of condensation, especially for low amplicon concentration templates. The incorporated optical system, using fluorescence response, could detect pathogen amplicon presence in collected samples, eliminating the need for gel electrophoresis. The system is therefore clinically relevant for DNA- or RNA-type sample analysis.

## Figures and Tables

**Table 1:** Heat extraction protocol reaction setup.

Solution Type	Target Gene	ExtractionParameters	Reverse Transcription Reagents	PCR Reagents
Cell Culture/ Cultured Viral Solution/ dH_2_O (NTC)	Nucleoprotein (601 bp)	70 °C/ 20 min 10 µL or 95 °C/ 10 min 10 µL or No heat 10 µL	▪ 2.0 µL of 10X RT Buffer ▪ 0.8 µL of 25X dNTP Mix (100 mM) ▪ 2.0 µL of R. primer 5′- GAACTCTGAGAACGAGCAAGA -3′ ▪ 1.0 µL of MultiScribe Reverse Transcriptase ▪ 10 µL of RNA sample ▪ 4.2 µL of Nuclease-free H_2_O	10 µL of Select Master Mix 1 µL of F. primer 5′- CTTGGAAGGTGATACCTCGTAAT -3′ 1 µL of R. primer 5′- GAACTCTGAGAACGAGCAAGA -3′ 3 µL of template (or 3 µL Nuclease-free H_2_O for NTC) 5 µL of deionized H_2_O
Cell Culture/ Cultured Viral Solution/ dH_2_O (NTC)	Spike (601 bp)	70 °C/ 20 min 10 µL or 95 °C/ 10 min 10 µL or No heat 10 µL	▪ 2.0 µL of 10X RT Buffer ▪ 0.8 µL of 25X dNTP Mix (100 mM) ▪ 2.0 µL of R. primer 5′- CAGTGCCAAGTCCAGAAGTAA -3′ ▪ 1.0 µL of MultiScribe Reverse Transcriptase ▪ 10 µL of RNA sample ▪ 4.2 µL of Nuclease-free H_2_O	10 µL of Select Master Mix 1 µL of F. primer 5′- CAAGCTGTTGTTGGTGCTATG -3′ 1 µL of R. primer 5′- CAGTGCCAAGTCCAGAAGTAA -3′ 3 µL of template (or 3 µL Nuclease-free H_2_O for NTC) 5 µL of deionized H_2_O

**Table 2:** RNA extraction comparison between protocols. The standard deviation is based on a set of three experiments.

Sample Type	Extraction Type	Before Extraction	After Extraction	A260/A280 (After Extraction)	Extraction Process Time	Extraction Cost
CC	Microprep Kit	32.9 ng/µL (SD = 4.96)	4.53 ng/µL (SD = 0.73)	1.88 (SD = 0.54)	45 to 75 min	$7 per reaction
	70 °C/20 min	38.7 ng/µL (SD = 6.38)	40.1 ng/µL (SD = 2.61)	0.84 (SD = 0.04)	20 min	≈$0
	95 °C/10 min	36.4 ng/µL (SD = 2.14)	39.5 ng/µL (SD = 7.72)	0.88 (SD = 0.04)	10 min	≈$0
CV	Microprep Kit	144 ng/µL (SD = 32.4)	10.8 ng/µL (SD = 10.7)	2.40 (SD = 0.66)	45 to 75 min	$7 per reaction
	70 °C/20 min	144 ng/µL (SD = 32.4)	224 ng/µL (SD = 42.5)	0.81 (SD = 0.01)	20 min	≈$0
	95 °C/10 min	144 ng/µL (SD = 32.4)	210 ng/µL (SD = 35.1)	0.88 (SD = 0.02)	10 min	≈$0

**Table 3:** C_T_ values comparison between protocols. The standard deviation is based on a set of three experiments.

Sample	Primer Type	Extraction Method	C_T_ Value	C_T_ Value Difference
CC	Nucleoprotein	Microprep Kit	33.13 (SD = 2.82)	0
		70 °C/20 min	36.57 (SD = 0.55)	+3.44
		95 °C/10 min	Undet *	Undet
	Spike	Microprep Kit	32.80 (SD = 1.02)	0
		70 °C/20 min	32.67 (SD = 1.35)	−0.13
		95 °C/10 min	32.76 (SD = 1.55)	−0.04
CV	Nucleoprotein	Microprep Kit	33.32 (SD = 0.45)	0
		70 °C/20 min	35.95 (SD = 0.77)	+2.63
		95 °C/10 min	36.44 (SD = 0.89)	+3.12
	Spike	Microprep Kit	32.29 (SD = 0.46)	0
		70 °C/20 min	30.69 (SD = 1.36)	−1.60
		95 °C/10 min	31.87 (SD = 0.97)	−0.42
NTC	Nucleoprotein	N/A	Undet	Undet
	Spike	N/A	Undet	Undet

* One reaction did not reach a C_T_ value within 40 cycles.

**Table 4:** Fluorescence measurement across both devices.

**Tube**	**Device Type**	**Fluorescence** **Values**	**Fluorescence** **Difference with NTC**
CC Spike 95 °C	StepOnePlus	21.2 (SD = 6.46)	−4.57
	Custom RT-PCR	445 (SD = 121)	+3.56
CC Nucleo 95 °C	StepOnePlus	15.0 (SD = 0.67)	+4.12
	Custom RT-PCR	545 (SD = 60.9)	−1.44
CV Spike 95 °C	StepOnePlus	22.0 (SD = 6.44)	−3.82
	Custom RT-PCR	523 (SD = 35.6)	+81.8
CV Nucleo 95 °C	StepOnePlus	18.0 (SD = 6.75)	+7.12
	Custom RT-PCR	574 (SD = 16.7)	+27.7
CC Spike 70 °C	StepOnePlus	20.3 (SD = 8.91)	−5.53
	Custom RT-PCR	444 (SD = 123)	+2
CC Nucleo 70 °C	StepOnePlus	11.4 (SD = 2.70)	+0.49
	Custom RT-PCR	506 (SD = 35.9)	−39.9
CV Spike 70 °C	StepOnePlus	17.1 (SD = 7.17)	−8.74
	Custom RT-PCR	525 (SD = 51.3)	+83.8
CV Nucleo 70 °C	StepOnePlus	14.7 (SD = 2.84)	+3.83
	Custom RT-PCR	611 (SD = 79.0)	+64.2
NTC Spike	StepOnePlus	25.8 (SD = 7.05)	0
	Custom RT-PCR	441 (SD = 79.3)	0
NTC Nucleo	StepOnePlus	10.9 (SD = 12.7)	0
	Custom RT-PCR	546 (SD = 42.1)	0

In red: negative difference; in blue: positive difference.

## Data Availability

Not applicable.

## Appendix A

Quick-RNA Microprep Kit Description [35].

- Product Contents
  ⚬ RNA Lysis Buffer
  ⚬ RNA Prep Buffer
  ⚬ RNA Wash Buffer (concentrate)
  ⚬ DNase/RNase-Free Water
  ⚬ DNase I^2^ (lyophilized)
  ⚬ DNA Digestion Buffer
  ⚬ Zymo-Spin™ IC Columns
  ⚬ Collection Tubes
  ⚬ Instruction Manual
- Specifications
  ⚬ Sample Sources—Cells (animal, gram(-) bacteria), soft and easy-to-lyse tissue, samples in DNA/RNA Shield™ or other preservation reagents, and enzymatic reactions (e.g., DNase I treated, Proteinase K treated). Not compatible with whole blood1 and urine1 samples.
  ⚬ Size—Total RNA including small/microRNAs (≥17 nt).
  ⚬ Purity—A260/A280 & A260/A230 > 1.8. RNA is ready for Next-Gen Sequencing, RT/qPCR, etc. Trace DNA can be removed by DNase I digestion.
  ⚬ Binding Capacity—Zymo-Spin™ IC Column yield up to 10 µg RNA.
  ⚬ Compatibility—For samples stored in preservation reagents: DNA/RNA Shield™, RNAprotect®, Allprotect®, Universal transport medium/viral transport medium (UTM®/VTM®), and RNAlater™.
  ⚬ Elution Volume—≥6 µL DNase/RNase-Free Water.
  ⚬ Equipment Needed (user provided)—Microcentrifuge, vortex.
- Protocol
  ⚬ Buffer Preparation
    ▪ Add 96 mL of 100% ethanol to the 24 mL of RNA Wash Buffer concentrate (R1050).
    ▪ Reconstitute lyophilized DNase I with DNase/RNase-Free Water, mix by gentle inversion, and store frozen aliquots: add 55 µL water.
  ⚬ Sample Preparation: Perform all steps at room temperature and centrifugation at 10,000–16,000× *g* for 30 s, unless specified.
    ▪ Remove cells liquid media from the culture container. Then add RNA Lysis Buffer directly to the monolayer. Remove cells from the culture surface by scraping, pipetting, etc.
  ⚬ Total RNA Purification: Perform all steps at room temperature and centrifugation at 10,000–16,000× *g* for 30 s, unless specified.
    1. Add 1 volume ethanol (95–100%) to 1 volume sample lysed in RNA Lysis Buffer (1:1) and mix well.
    2. Transfer the mixture into a Zymo-Spin™ IC Column in a Collection Tube and centrifuge. Discard the flow-through.
    3. DNase I treatment (recommended)
      - Wash the column with 400 µL of RNA Wash Buffer and centrifuge. Discard the flowthrough.
      - In a nuclease-free tube, add 5 µL of DNase I (1 U/µL) and 35 µL of DNA Digestion Buffer and mix. Add mixture directly into the column matrix.
      - Incubate the column at room temperature (20–30 °C) for 15 min.
    4. Add 400 µL of RNA Prep Buffer to the column and centrifuge. Discard the flow-through.
    5. Add 700 µL of RNA Wash Buffer to the column and centrifuge. Discard the flow-through.
    6. Add 400 µL of RNA Wash Buffer and centrifuge the column for 1 min to ensure complete removal of the wash buffer. Then carefully, transfer the column into a nuclease-free tube (not provided).
    7. Add 15 µL of DNase/RNase-Free Water directly to the column matrix and centrifuge.

## References

[1] World Health Organization Who Coronavirus (COVID-19) Dashboard. World Health Organization. https://covid19.who.int/.

[2] Pritt B.S., Wang P., Nuzzo J., Zimmermann S., Burnham C.-A.D. (2021). Deadly pathogens, transformative technologies, and protracted pandemics: Challenges and opportunities in laboratory medicine. Clin. Chem..

[3] Bong C.-L., Brasher C., Chikumba E., McDougall R., Mellin-Olsen J., Enright A. (2020). The COVID-19 pandemic: Effects on low- and middle-income countries. Anesth. Analg..

[4] Zhu H., Zhang H., Xu Y., Laššáková S., Korabečná M., Neužil P. (2020). PCR past, present and future. BioTechniques.

[5] Garibyan L., Avashia N. (2013). Polymerase chain reaction. J. Investig. Dermatol..

[6] Wright W.F., Simner P.J., Carroll K.C., Auwaerter P.G. (2021). Progress report: Next-generation sequencing (NGS), multiplex polymerase chain reaction (PCR), and broad-range molecular assays as diagnostic tools for fever of unknown origin (FUO) investigations in adults. Clin. Infect. Dis..

[7] Preston C.M., Harris A., Ryan J.P., Roman B., Marin R., Jensen S., Everlove C., Birch J., Dzenitis J.M., Pargett D. (2011). Underwater Application of quantitative PCR on an ocean mooring. PLoS ONE.

[8] Liu P., Seo T.S., Beyor N., Shin K.-J., Scherer J.R., Mathies R.A. (2007). Integrated portable polymerase chain reaction-capillary electrophoresis microsystem for rapid forensic short tandem repeat typing. Anal. Chem..

[9] Noviyanti F., Shimizu S., Hosotani Y., Koseki S., Inatsu Y., Kawasaki S. (2020). Predictive growth model of listeria monocytogenes under fluctuating temperature conditions in pasteurized milk by using real-time polymerase chain reaction. Foodborne Pathog. Dis..

[10] Shafi A., Farooq U., Akram K., Khan M.Z., Hayat Z., Hayat K. (2021). Molecular epidemiology of foodborne diseases. Sequencing Technologies in Microbial Food Safety and Quality.

[11] Prevalence of Private Drinking Water Wells is Associated with Salmonellosis Incidence in Maryland, USA: An Ecological Analysis Using Foodborne Diseases Active Surveillance Network (FoodNet) Data (2007–2016). https://www.researchgate.net/publication/351506261_Prevalence_of_Private_Drinking_Water_Wells_is_Associated_with_Salmonellosis_Incidence_in_Maryland_USA_An_Ecological_Analysis_Using_Foodborne_Diseases_Active_Surveillance_Network_FoodNet_Data_2007-2016.

[12] Chan K., Wong P.-Y., Yu P., Hardick J., Wong K.-Y., Wilson S.A., Wu T., Hui Z., Gaydos C., Wong S.S. (2016). A rapid and low-cost PCR thermal cycler for infectious disease diagnostics. PLoS ONE.

[13] A Micromachined Low-Power-Consumption Portable PCR System. https://www.researchgate.net/publication/267721096_A_Micromachined_Low-power-consumption_Portable_PCR_System.

[14] Schneegaß I., Bräutigam R., Köhler J.M. (2001). Miniaturized flow-through PCR with different template types in a silicon chip thermocycler. Lab Chip.

[15] Hennig M., Braun D. (2005). Convective polymerase chain reaction around Micro Immersion Heater. Appl. Phys. Lett..

[16] Oda R.P., Strausbauch M.A., Huhmer A.F., Borson N., Jurrens S.R., Craighead J., Wettstein P.J., Eckloff B., Kline B., Landers J.P. (1998). Infrared-mediated thermocycling for ultrafast polymerase chain reaction amplification of DNA. Anal. Chem..

[17] Kadja T., Liu C., Sun Y., Chodavarapu V.P. (2022). Low-cost, real-time polymerase chain reaction system for point-of-care medical diagnosis. Sensors.

[18] Center for Food Safety and Applied Nutrition Listeria (listeriosis). U.S. Food and Drug Administration. https://www.fda.gov/food/foodborne-pathogens/listeria-listeriosis#:~:text=Listeria%20monocytogenes%20(L.,and%20other%20food%20preservation%20measures.

[19] Komiazyk M., Walory J., Kozinska A., Wasko I., Baraniak A. (2021). Impact of the nucleic acid extraction method and the RT-qpcr assay on SARS-COV-2 detection in low-viral samples. Diagnostics.

[20] Kralik P., Ricchi M. A Basic Guide to Real Time PCR in Microbial Diagnostics: Definitions, Parameters, and Everything. https://www.frontiersin.org/articles/10.3389/fmicb.2017.00108/full.

[21] Lõoke M., Kristjuhan K., Kristjuhan A. (2017). Extraction of genomic DNA from yeasts for PCR-based applications. BioTechniques.

[22] de Almeida I.N., da Silva Carvalho W., Rossetti M.L., Costa E.R., de Miranda S.S. (2013). Evaluation of six different DNA extraction methods for detection of mycobacterium tuberculosis by means of PCR-IS6110: Preliminary study. BMC Res. Notes.

[23] Zarzoso-Lacoste D., Corse E., Vidal E. (2012). Improving pcr detection of prey in molecular diet studies: Importance of group-specific primer set selection and Extraction Protocol performances. Mol. Ecol. Resour..

[24] Heller L.C., Davis C.R., Peak K.K., Wingfield D., Cannons A.C., Amuso P.T., Cattani J. (2003). Comparison of methods for DNA isolation from food samples for detection of shiga toxin-producing Escherichia coli by real-time PCR. Appl. Environ. Microbiol..

[25] Tell L.A., Foley J., Needham M.L., Walker R.L. (2003). Comparison of four rapid DNA extraction techniques for conventional polymerase chain reaction testing of three mycobacterium spp. that affect birds. Avian Dis..

[26] Elizaquível P., Aznar R. (2008). Comparison of four commercial DNA extraction kits for PCR detection of listeria monocytogenes, salmonella, Escherichia coli O157:H7, and Staphylococcus aureus in fresh, minimally processed vegetables. J. Food Prot..

[27] Wang T.Y., Wang L., Zhang J.H., Dong W.H. (2011). A simplified universal genomic DNA extraction protocol suitable for PCR. Genet. Mol. Res..

[28] Barza R., Patel P., Sabatini L., Singh K. (2020). Use of a simplified sample processing step without RNA extraction for direct SARS-COV-2 RT-PCR detection. J. Clin. Virol..

[29] Pastorino B., Bessaud M., Grandadam M., Murri S., Tolou H.J., Peyrefitte C.N. (2005). Development of a taqman® RT-PCR assay without RNA extraction step for the detection and quantification of African chikungunya viruses. J. Virol. Methods.

[30] Camacho-Sanchez M., Burraco P., Gomez-Mestre I., Leonard J.A. (2013). Preservation of RNA and DNA from mammal samples under field conditions. Mol. Ecol. Resour..

[31] Chibo D., Birch C. (2006). Analysis of human coronavirus 229e spike and nucleoprotein genes demonstrates genetic drift between chronologically distinct strains. J. Gen. Virol..

[32] Reverse Transcription Setup: Thermo Fisher Scientific—US. https://www.thermofisher.com/us/en/home/life-science/cloning/cloning-learning-center/invitrogen-school-of-molecular-biology/rt-education/reverse-transcription-setup.html.

[33] King A. (2012). In Virus Taxonomy: Classification and Nomenclature of Viruses.

[34] Fenwick C., Croxatto A., Coste A.T., Pojer F., André C., Pellaton C., Farina A., Campos J., Hacker D., Lau K. (2021). Changes in SARS-COV-2 spike versus nucleoprotein antibody responses impact the estimates of infections in population-based seroprevalence studies. J. Virol..

[35] Quick-RNA Microprep Kit—ZYMO Research. https://files.zymoresearch.com/protocols/_r1050_r1051_quick-rna_microprep_kit.pdf.

